# Transpirational cooling enhances grain yield and quality in heat-tolerant rice varieties

**DOI:** 10.3389/fpls.2025.1660130

**Published:** 2025-10-06

**Authors:** Qilin Mu, Song Wang, Yangxuan Liu, Yuan Gao, Wenyan Liu, Nnaemeka Emmanuel Okpala

**Affiliations:** ^1^ MARA Key Laboratory of Sustainable Crop Production in the Middle Reaches of the Yangtze River (Co-construction by Ministry and Province)/Hubei Key Laboratory of Waterlogging Disaster and Agricultural Use of Wetland, College of Agriculture, Yangtze University, Jingzhou, China; ^2^ Hubei Key Laboratory of Food Crop Germplasm and Genetic Improvement/Institute of Food Crops, Hubei Academy of Agricultural Sciences, Wuhan, China; ^3^ Hubei Collaborative Innovation Center for Grain Industry/College of Agriculture, Yangtze University, Jingzhou, China

**Keywords:** heat stress, grain filling, stomata, head rice rate, grain weight

## Abstract

High temperatures during the grain-filling stage in rice often shorten grain-filling duration and accelerate maturity, typically reducing grain yield and quality. Leaf evaporative cooling via transpiration has been identified as an important adaptive mechanism for heat stress in rice. However, its relevance in evaluating varietal tolerance to heat during grain filling remains unclear. In this study, we assessed 28 newly bred, high-quality rice varieties to examine the extent of, and genotypic variation in, transpirational cooling and its effects on grain yield and quality under a staggered sowing system (S1 and S2). Our results showed that high temperatures significantly decreased 1,000-grain weight by 1.6 g and head rice rate by 6.7%, while increasing chalkiness by 3.3%. Cluster analysis grouped the varieties into three categories of heat tolerance: tolerant, intermediate, and susceptible. Under heat stress, heat-tolerant varieties maintained significantly lower leaf and panicle temperatures and exhibited higher stomatal conductance than heat-susceptible varieties during grain filling. These tolerant varieties also possessed higher stomatal density and total stomatal area. Furthermore, the change in 1,000-grain weight was negatively correlated with stomatal density, whereas the change in head rice rate showed a negative correlation with stomatal size. These findings suggest that varietal differences in heat tolerance during grain filling may parallel those observed at anthesis. Stomatal regulation of the canopy microenvironment may serve as a general physiological basis for varietal heat response in rice.

## 1 Introduction

Advances in rice breeding have led to sustained improvements in yield and grain quality. As China develops into a modern society as a whole, consumers are increasingly demanding rice with better grain quality (Liu et al., 2020b; Yan et al., 2021; Calingacion et al., 2014). A growing number of scientists have also begun to focus on improving crop grain quality rather than solely concentrating on yield (Zhang et al., 2016; Lin et al., 2020; Yang et al., 2024; Lu et al., 2020). On the other hand, agricultural departments of governments have allocated more projects and funds to rice quality enhancement in recent years. For instance, as early as 2021, Hubei Province in China identified the development of high-quality rice as the top priority among its ten key agricultural industrial chains. This indicates that the rice industry has entered an era of quality-oriented development.

A crucial growth stage in rice is the ripening or grain-filling stage, which usually lasts 30–40 days and largely determines both rice yield and grain quality (Liu et al., 2019a). However, more studies in rice heat stress to date have focused on the heading stage rather than the grain-filling stage (Matsui et al., 2001; Jagadish et al., 2010; Julia and Dingkuhn, 2013). Typically, a daily average temperature above 30 °C or a daily maximum temperature above 35 °C during the heading and flowering stage is considered high temperature, whereas temperatures below these thresholds are regarded as normal (Liu et al., 2019b). During the grain-filling stage, a daily average temperature above 28 °C is classified as high temperature, while a range of 26 °C–28 °C is considered normal temperature.

Previous studies have shown that heat-forced ripening significantly increased chalkiness and reduced head rice rate (Deng et al., 2015; Nagato and Ebata, 1965; Yoshida and Hara, 1977; Tashiro and Wardlaw, 1991; Kazuo et al., 2001; Wakamatsu et al., 2007). Comparative analyses of rice varieties grown in dry and rainy seasons showed that high temperatures combined with high humidity generally increased chalkiness in the rainy season–despite only slight differences in temperature between seasons—compared with the dry season (Zhao and Fitzgerald, 2013). Jagadish et al. noted that three factors–the source–sink relationship, the sink activity, and hormonal balance—might be responsible for quality loss and increased chalk formation in rice grown under heat stress (Jagadish et al., 2015). Similarly, Bahuguna observed that rice varieties with contrasting heat tolerance levels respond differently to the three factors, and as a result, the impact of heat stress to their grain quality also varies (Bahuguna et al., 2017).

Scientists have long recognized the special role of stomata in rice under heat stress. Zhang et al. (2007) noted that these variations might be directly related to stomatal conductance. It has been reported that heat stress reduces stomatal conductance, leading to lower CO_2_ assimilation and water loss (Taratima et al., 2022). There is a correlation between stomatal conductance and photosynthetic efficiency (Alhaithloul, 2019). A decrease in stomatal conductance is one of the adaptive mechanisms that plants use to survive heat stress (Giri et al., 2017; Wu et al., 2018). Lo et al. (2020) showed that an increase in stomatal conductance due to high temperature in maize can increase transpiration rate. According to Jiang et al. (2023), transpiration is an important indicator of lower surface temperature in rice plants, as the evaporation of water transfers heat away from the plant surface. Yoshimoto et al. (2022) also observed that transpiration can lead to evaporative cooling in rice. Therefore, an increase in stomatal conductance can be a strategy for heat tolerance in rice. Stomata, mainly located in the leaves, regulate plant temperature and canopy microenvironmental conditions. During heat stress, plants undergo anatomical adaptations that alter stomatal numbers on the leaf surface (Taratima et al., 2022). Stomatal responses to heat may be a key factor in determining how rice varieties respond to heat stress (Casson and Gray, 2008). Taratima et al. (2022) found significant differences in stomatal length in rice seedlings grown under heat stress. A reduction in stomatal density has also been observed in rice exposed to heat stress (Kondamudi et al., 2012). However, it has been reported that plants do not rely on increased stomatal density to overcome heat stress (Taratima et al., 2022), but instead employ other mechanisms such as increasing stomatal pore size or pore openings (Zhou et al., 2016), which in turn lead to higher stomatal conductance and photosynthesis efficiency (Taratima et al., 2022).

Meanwhile, heat-forced ripening usually has a lower threshold temperature than heat stress during heading (Nevame et al., 2018). Heat stress during heading mainly involves the anther and pollen, with an effective duration of approximately one week, during which rice completes flowering and pollination. In contrast, heat-forced ripening involves florets and grains, with an effective duration of approximately a month or more, during which grain filling is completed. This process involves the production and transport of carbohydrates, as well as starch accumulation (Yamakawa and Hakata, 2010; Zhang et al., 2018). It has not been established whether rice varietal differences in response to heat stress is associated with stomatal conductance at grain filling, as they are at heading. The aim of this study was to investigate the impact of transpirational cooling in different rice varieties during heat-forced ripening and to establish possible varietal differences in stomatal conductance and stomatal structural characteristics.

## 2 Materials and methods

### 2.1 Field experiment and rice cultivation

The experiment was carried out in 2019 at the Experimental Farm of Yangtze University in Jingzhou, Hubei Province, China. The soil texture at the experimental site was clay loam, with 18.3 g/kg organic matter, 1.14 g/kg hydrolyzable N, 34.25 mg/kg available P, and 105.13 mg/kg available K.

A staggered sowing method was used for the experiment, conducted in two different batches: Batch 1 (Crop1) and Batch 2 (Crop2). Meanwhile, small adjustments in sowing time were made so that all tested rice varieties headed and flowered at approximately the same time within the same batch, ensuring that the temperature during grain filling was nearly the same for all the varieties.

The experiment was conducted in randomized blocks, with each plot measuring 11.2 m^2^, 20 cm row spacing, and each treatment replicated three times per season. A single basal application of compound fertilizer (N:P_2_O_5_:K_2_O = 22:8:12, 150 kg/1,000 m^2^) was applied to each plot.

### 2.2 Observations and determinations

#### 2.2.1 Meteorological observations

The meteorological data, including daily mean temperature, daily maximum temperature, daily relative humidity, insolation hours, and daily rainfall, were obtained from a state-standard meteorological station, the Jingzhou Agricultural Meteorological Experimental Station, located approximately 500 m from the experimental field.

#### 2.2.2 Rice varieties

A total of 28 high-quality rice varieties were used in the experiment, all newly developed in southern China in recent years and including both conventional and hybrid types.

#### 2.2.3 Crop phenology

Before heading, rice phenology was monitored daily in the field at 17:00 h. At the onset of heading, the heading date of each rice variety was recorded. The heading dates were determined by the 1.5 cm emergence of the neck-panicle node from the leaf sheath. The initial heading stage was defined as a 10% heading rate within the plot, while the full heading stage was defined as an 80% heading rate.

#### 2.2.4 Grain weight and quality

The filled rice grains were dried in the sun to a moisture content of approximately 13.5%. The head rice rate, chalkiness, chalky grain rate, and 1,000-grain weight were measured according to the *Standard of Quality of Edible Rice Varieties* (NY/T 593-2013) of the Chinese Ministry of Agriculture and Rural Affairs.

#### 2.2.5 Measurement of stomatal density and size

The stomatal density and size were determined after flag leaves had fully unfolded for each variety. At 09:00, three flag leaves of the same physiological age were selected. The modified scraping method was used to treat the test material (Radoglou and Jarvis, 1990). The leaves were cut along the leaf sheath, and the middle part of each leaf (approximately 10 cm in length) was transferred into a centrifuge tube containing distilled water, labeled, and stored at 4 °C. For stomatal density, the number of stomata (No.) was first calculated using five visual fields under an upright phase-contrast microscope imaging system/CI-E (Nikon, Japan) with a 10 × 20 lens (actual area of the region: 0.33 mm^2^). In addition, 10 visual fields were randomly photographed under a 20 × 20 lens, with 10 stomata in each field. Afterwards, the length (L) and width (W) of the stomata were measured. Statistical analyses were performed using the software ImageJ (National Institutes of Health, USA). The calculation method for stomatal size (S) was similar to that adopted by Zhang et al. (2019), being:


$$\text{S}=\frac{\text{L}}{2}\times \frac{\text{W}}{2}\times \text{π }(\mu {\text{m}}^{2})$$

where length was the stomatal length of the dumbbell-shaped stomatal apparatus, and width was the widest point perpendicular to the dumbbell-shaped stomatal apparatus (Li et al., 2010).

The stomatal density was divided into two categories: quantity density (QD), which was defined as stomata number on per unit leaf area, being:


$$\text{QD}=\frac{\text{No}.}{0.33}\;(\text{No}.{\text{ mm}}^{-2})$$

and the area density (AD), which was defined as the sum of each stomatal area on per unit leaf area, being:


$$\text{AD}=\text{QD}\times \text{S }{(\text{μm}}^{2}{\text{mm}}^{-2})$$

#### 2.2.6 Stomatal conductance

Four rice varieties from the 28, with varying heat tolerance levels–Taiyou 98 and Wanxiangyou 982 (heat-tolerant, *indica* hybrid rice), and Shuijing 3 and Shennongyou 228 (heat-susceptible, *japonica* hybrid rice)–were selected to determine stomatal conductance. In other to ensure that the flag leaves were at the same physiological age, protruding panicles were marked at the onset of the heading stage and selected for the stomatal conductance determination. From three days after full heading to 20 days thereafter, flag leaves from the marked plants were selected for stomatal conductance measurement in triplicate every three days for each variety using a portable photosynthesis meter (LI-6400XT,LI Co.ld, USA) between 10:00 h and 11:00 h.

#### 2.2.7 Leaf temperature and panicle temperature

Flag leaf and panicle temperature were determined using a handheld infrared thermometer (MI-230, Apogee Instruments) at 10:00 h and 14:00 h, respectively. For each variety, five flag leaves and five panicles with the same physiological age were used at each measurement. Measurements were conducted at 2, 5, and 7 days after heading (DAH).

#### 2.2.8 Correlation analysis of stomatal characteristics and heat tolerance

The differences (deltas) in chalkiness, milled rice rate, head rice rate, and 1,000-grain weight between S2 and S1 were used to express the thermal sensitivity of each rice variety. The correlations between each delta and stomatal characteristics (size, density, and area) were examined.

### 2.3 Varietal difference cluster and data analyses

A cluster analysis of varietal responses to heat during grain filling was carried out using the head rice rate delta and grain weight delta between high temperature (S2) and normal temperature (S1) as benchmarks for rice variety sensitivity to high temperature. DPS 7.5 software was used for the statistical analyses. For the cluster analysis, hierarchical clustering with Euclidean distance and the single linkage method was employed.

## 3 Results

### 3.1 Weather profile and weather conditions experienced by rice in different cultivation seasons

**Figure 1:**
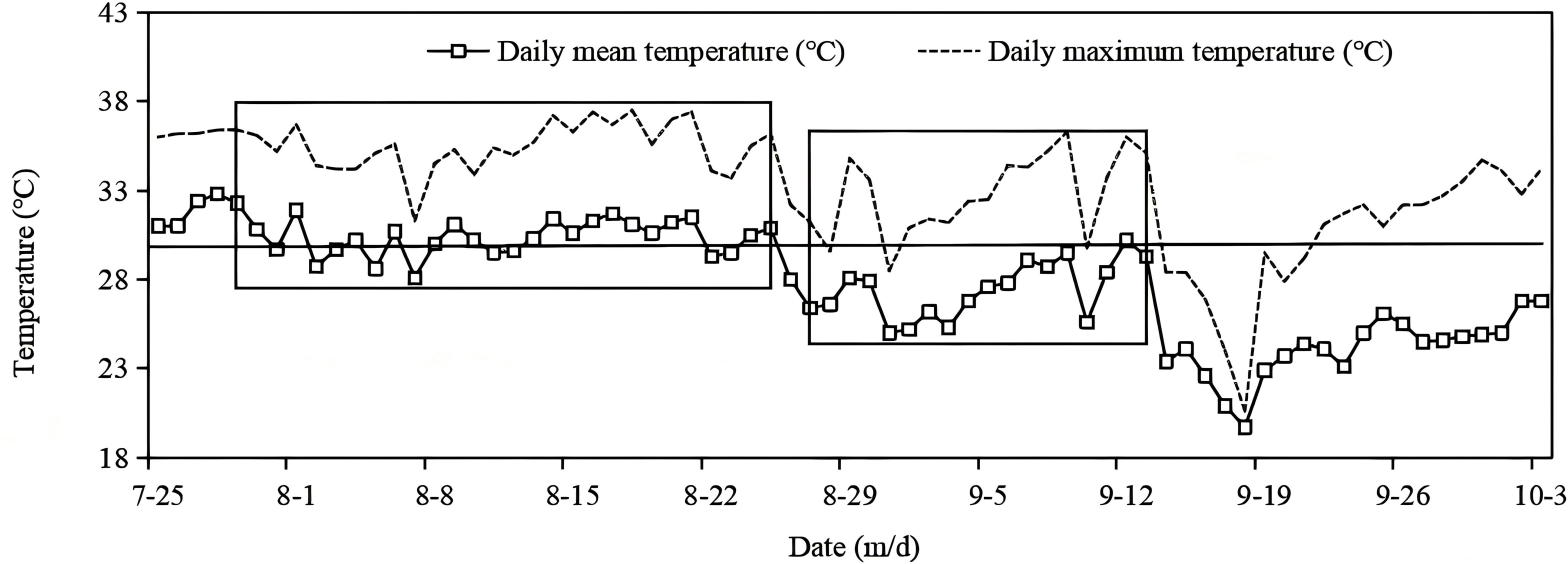
Temperature trend from heading to grain filling in rice at the Yangtze University experimental farm in 2019. The additional boxes indicate the time period of the grain filling of the two batches of sown rice.

During the rice growing season, high temperatures occurred from late July to late August, followed by a moderately hot period from late August to 14 September. Subsequently, then the weather entered into a normal-temperature period, which coincided with grain filling from mid-September to early October (**Figure 1**). As shown in **Table 1**, most varieties in S1 flowered from early to mid-August, whereas most varieties in S2 flowered in early September. The duration of grain filling ranged from 30 to 35 days in S1 and from 35 to 45 days in S2. Thus, in S1, grain filling mostly took place during high-temperature periods, whereas in S2 it mostly occurred during normal-temperature conditions.

**Table 1:** Heading dates of 28 rice varieties under a staggered sowing system treatment (S1 and S2).

Varieties	Initial heading date	
	S1	S2
Qunyou 1256	6-Aug	6-Sep
Yexiangyousimiao	6-Aug	3-Sep
Shuijing 3	12-Aug	27-Sep
Qunyou 2	8-Aug	4-Sep
Yuzhenxiang	3-Aug	1-Sep
Huruan 1212	21-Aug	7-Sep
Chuanyou6203	5-Aug	5-Sep
Yexiangyouxinhuazhan	5-Aug	3-Sep
Xiligongmi	15-Aug	13-Sep
Changtianyou 9	2-Aug	31-Aug
Baixiangyou 9978	8-Aug	5-Sep
Baixiangyou 125	8-Aug	5-Sep
Shennongyou 228	8-Aug	4-Sep
Guiyu 8	8-Aug	6-Sep
Yexiangyoulisi	6-Aug	5-Sep
Yujing 91	4-Aug	1-Sep
Taixiangyouyuesimiao	12-Aug	10-Sep
Yuxiang 203	15-Aug	9-Sep
Taiyou 398	29-Jul	30-Sep
Guiyu 9	8-Aug	7-Sep
Nongxiang 32	5-Aug	5-Sep
Exiang 2	23-Aug	13-Sep
Wanxiangyou 982	12-Aug	6-Sep
Taoyouxiangzhan	6-Aug	1-Sep
Taiyou 871	12-Aug	5-Sep
Yixiangyou 2115	13-Aug	5-Sep
Taiyou 98	6-Aug	4-Sep
Jiafengyou 2	25-Aug	7-Sep

### 3.2 Effect of different weather conditions on grain weight and rice quality

High temperature significantly reduced grain weight and the main quality indicators of each variety compared to normal temperature (**Table 2**). Our results showed that among the 28 varieties tested, high temperature significantly reduced 1,000-grain weight in 24 varieties, with an average of 1.6 g. Similarly, high temperature significantly reduced the head rice rate in 20 varieties and increased chalkiness in 12 varieties and the chalky kernel rate in nine varieties, with average changes of 6.7%, 3.3%, and 10.3%, respectively.

**Table 2:** Rice quality and 1,000-grain weight of 28 rice varieties under high temperature versus normal temperature.

Varieties	Chalkiness		Chalky grain rate (%)		Head rice rate (%)		1,000-grain weight (g)	
	S1	S2	S1	S2	S1	S2	S1	S2
Qunyou 1256	16.9	3.5**	37.8	10.3**	45.7	59.2**	20.9	23.3**
Yexiangyou simiao	0.9	0.1	4.1	1.0	58.2	68.5**	18.7	20.9**
Shuijing 3	6.4	0.6**	20.2	1.1**	58.8	67.6**	23.5	26.2**
Qunyou 2	3.4	1.5**	12.4	5.5**	52.8	60.0**	19.8	22.3**
Yuzhenxiang	0.8	0.1	2.2	0.1	48.5	55.5**	26.5	29.5**
Huruan 1212	8.3	3.7**	24.9	15.8	58.1	59.9	20.0	23.6**
Chuanyou 6203	2.7	0.7**	6.6	3.6	52.0	62.1**	27.5	27.9
Yexiangyouxinhuazhan	0.9	0.3	1.9	1.0*	61.5	67.3**	19.9	20.3
Xiligongmi	1.2	0.8	3.6	2.8	56.8	61.2**	22.8	23.6**
Changtianyou 9	2.2	0.4**	9.1	2.0**	49.6	59.2**	21.8	23.3**
Baixiangyou 9978	1.7	0.3**	3.2	0.5	59.8	68.7**	22.2	23.9**
Baixiangyou 125	1.7	0.1**	3.0	0.3	55.2	64.1**	17.8	19.1**
Shennongyou 228	3.5	1.4**	12.6	5.8**	52.9	60.8**	21.5	22.9**
Guiyu 8	1.0	0.3	2.2	1.1	59.4	66.9**	20.1	21.7**
Yexiangyou lisi	0.5	0.1	1.7	0.3	58.7	66.0**	18.9	20.1**
Yujing 91	0.4	0.1	1.4	0.3	53.7	60.1**	31.8	33.2**
Taixiangyou yuesimiao	1.5	0.8	6.6	3.0*	60.3	64.1**	23.1	24.4**
Yuxiang 203	4.9	1.1	17.7	4.7**	53.5	57.0*	28.0	29.4**
Taiyou 398	1.0	0.5	3.6	1.9	61.3	64.2*	22.0	23.5**
Guiyu 9	0.5	0.1	2.4	0.3	60.6	63.4*	21.9	23.6**
Nongxiang 32	0.3	0.2	1.5	1.4	56.6	59.3	25.1	27.1**
Exiang 2	1.0	0.3	2.7	1.4	61.2	62.3	27.7	29.3**
Wanxiangyou 982	2.6	0.5**	9.5	2.0**	57.8	61.0*	22.1	23.8**
Taoyouxiangzhan	0.7	0.3	1.8	1.5	59.6	60.3	26.4	27.8**
Taiyou 871	2.1	0.6**	4.5	1.4	57.4	59.8	23.4	23.8
Yixiangyou 2115	2.3	1.7	9.9	6.8	59.4	60.7	33.6	33.9
Taiyou 98	1.4	0.8	3.3	2.2	65.9	66.4	23.8	24.4**
Jiafengyou 2	2.4	0.6**	6.9	1.5	61.7	60.1	22.9	23.9**

* and ** indicate significant differences between C1 and C2 for each variety at P<0.05 and P<0.01, respectively.

### 3.3 Classification of varieties by their responses to heat stress during grain filling

**Figure 2:**
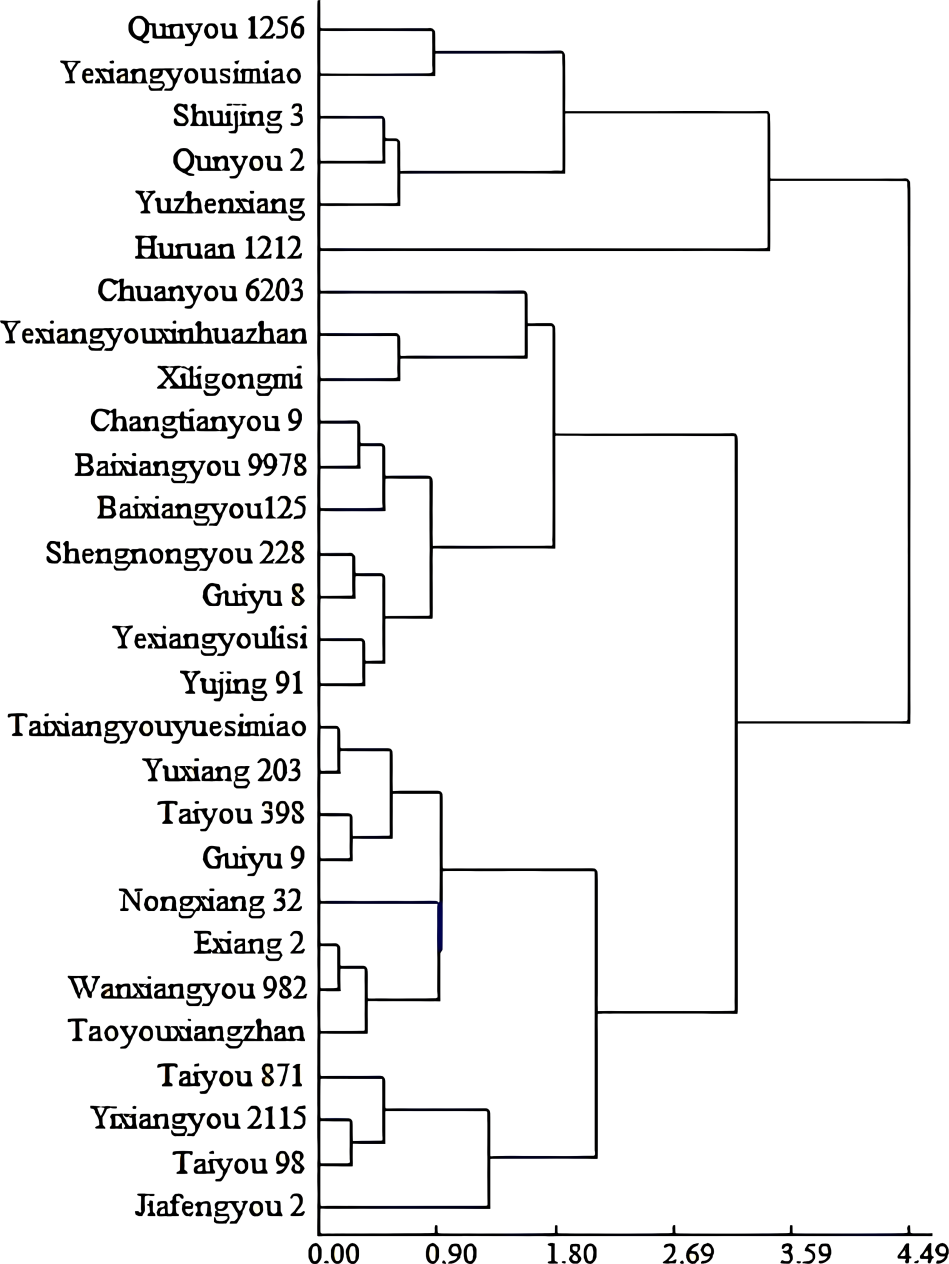
Classification of rice varieties with contrasting heat tolerances based on their delta-value for 1,000-grain weight and head rice rate, under high temperature vs normal temperature, using a clustering analysis.

The deltas for 1,000-grain weight and head rice rate of all varieties under high and normal temperature conditions were used as varietal response indicators. Based on these two factors, a cluster analysis was conducted, which divided the 28 varieties into two major groups: six heat-susceptible varieties and 22 heat-tolerant varieties. Because the latter group was too broad in varietal composition, it was further divided into two subgroups: the heat-intermediate group, including 10 varieties, and the heat-tolerant group, including 12 varieties (**Figure 2**).

### 3.4 Differences in leaf temperature, panicle temperatures, and stomatal conductance in four selected varieties

**Figure 3:**
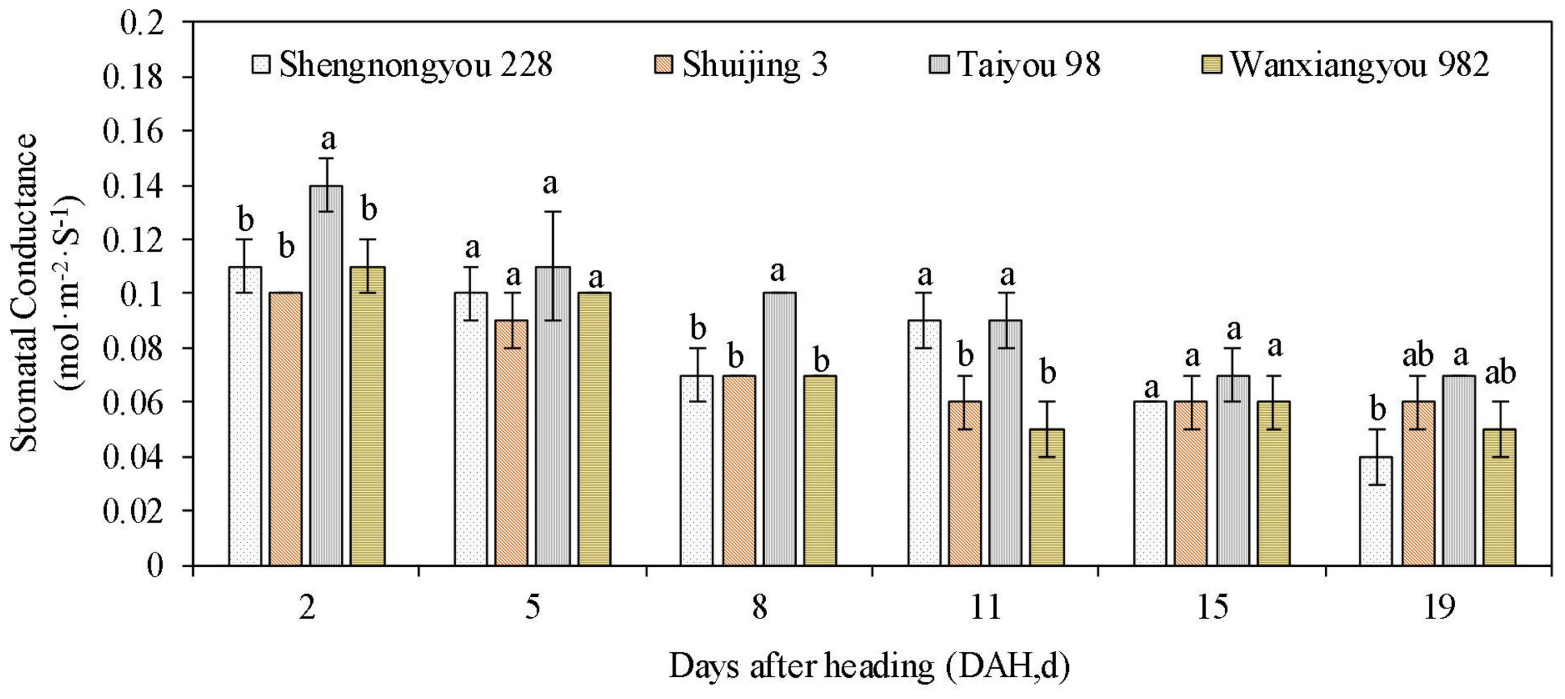
Stomatal conductance of rice varieties after heading. Here, DAH indicates the determining dates at some intervals after panicle heading. Determination was conducted each morning between 10:00 and 11:00. Different lowercase letters above each bar represent statistically significant differences (P<0.05) between varieties. Heat tolerance types for varieties: Taiyou 98 and Wanxiangyou 982 belong to the tolerant group, Shuijing 3 the susceptible group, and Shennongyou 228 the intermediate group based on the clustering analysis (**Figure 2**), respectively.

Leaf and panicle temperatures of heat-tolerant varieties were significantly lower than those of susceptible varieties in the morning (10:00) and afternoon (14:00, the time of peak air temperature) (**Table 3**). We also observed that the difference between leaf and panicle temperatures in heat-susceptible varieties remained significantly high, whereas the difference in heat-tolerant varieties was low. Similarly, the stomatal conductance of heat-tolerant varieties remained higher than that of susceptible varieties during most periods after heading (**Figure 3**).

**Table 3:** Monitoring of leaf and panicle temperatures during grain filling.

DAH	Time	Variety	Temperature (°C)		
			Leaf	Panicle	Panicle-leaf
2	10:00	Shennongyou228	32.9 ± 0.1 a	34.8 ± 0.2 a	1.9 a
		Shuijing 3	33.1 ± 0.2 a	34.7 ± 0.6 ab	1.6 ab
		Taiyou98	32.5 ± 0.2 b	33.33 ± 0.1 c	0.8 b
		Wanxiangyou982	32.3 ± 0.1 b	34.0 ± 0.5 bc	1.7 a
	14:00	Shennongyou228	35.4 ± 0.2 a	37.3 ± 0.4 a	1.9 ab
		Shuijing 3	35.3 ± 0.2 a	37.8 ± 0.3 a	2.6 a
		Taiyou98	33.3 ± 0.0 b	35.0 ± 0.4 b	1.7 b
		Wanxiangyou982	32.6 ± 0.1 c	34.0 ± 0.1 c	1.5 b
5	10:00	Shennongyou228	31.5 ± 0.2 a	33.5 ± 0.1 a	2.0 a
		Shuijing 3	31.0 ± 0.1 b	32.5 ± 0.1 b	1.5 ab
		Taiyou98	31.0 ± 0.2 c	31.4 ± 0.1 c	0.8 c
		Wanxiangyou982	30.5 ± 0.3 c	31.6 ± 0.2 d	1.2 bc
	14:00	Shennongyou228	35.5 ± 0.3 a	36.7 ± 0.0 b	1.2 b
		Shuijing 3	35.0 ± 0.3 a	38.0 ± 0.2 a	2.0 a
		Taiyou98	34.4 ± 0.1 b	35.9 ± 0.1 c	1.4 b
		Wanxiangyou982	33.6 ± 0.2 c	35.8 ± 0.0 c	2.3 a
7	10:00	Shennongyou228	31.3 ± 0.1 b	33.0 ± 0.3 a	1.7 a
		Shuijing 3	31.6 ± 0.1 a	33.2 ± 0.1 a	1.6 a
		Taiyou98	31.2 ± 0.1 b	32.6 ± 0.0 b	1.4 ab
		Wanxiangyou982	31.3 ± 0.1 b	32.3 ± 0.1 b	1.1 b
	14:00	Shennongyou228	32.1 ± 0.1 ab	33.0 ± 0.0 ab	0.9 a
		Shuijing 3	32.2 ± 0.2 a	33.2 ± 0.1 a	1.0 a
		Taiyou98	32.0 ± 0.1 b	32.7 ± 0.2 bc	0.7 a
		Wanxiangyou982	32.0 ± 0.1 b	32.5 ± 0.2 c	0.6 a

Data with different lowercase letters represent statistically significant differences (P<0.05) between varieties at the same observational time of day. Heat tolerance types for varieties: Taiyou 98 and Wanxiangyou 982 belongs to the tolerant group, Shuijing 3 the susceptible group and Shennongyou 228 the intermediate group, based on the clustering analysis (**Figure 2**), respectively.

**Table 3** presents leaf and panicle temperatures during grain filling. Data with different lowercase letters indicate statistically significant differences (P<0.05) between varieties at the same time of day. Heat tolerance classifications were as follows: Taiyou 98 and Wanxiangyou 982 belonged to the tolerant group, Shuijing 3 to the susceptible group, and Shennongyou 228 to the intermediate group, based on the clustering analysis (**Figure 2**).

**Figure 3** presents the stomatal conductance of rice varieties after heading. Here, DAH refers to days after heading, measured at specific intervals following panicle interval. Measurements were taken each morning between 10:00 and 11:00. Different lowercase letters above each bar indicate statistically significant differences (P<0.05) between varieties. Heat tolerance classifications were as follows: Taiyou 98 and Wanxiangyou 982 belonged to the tolerant group, Shuijing 3 to the susceptible group, and Shennongyou 228 to the intermediate group, based on the clustering analysis (**Figure 2**), respectively.

### 3.5 The effect of stomatal density and size on varietal heat stress tolerance difference

**Figure 4:**
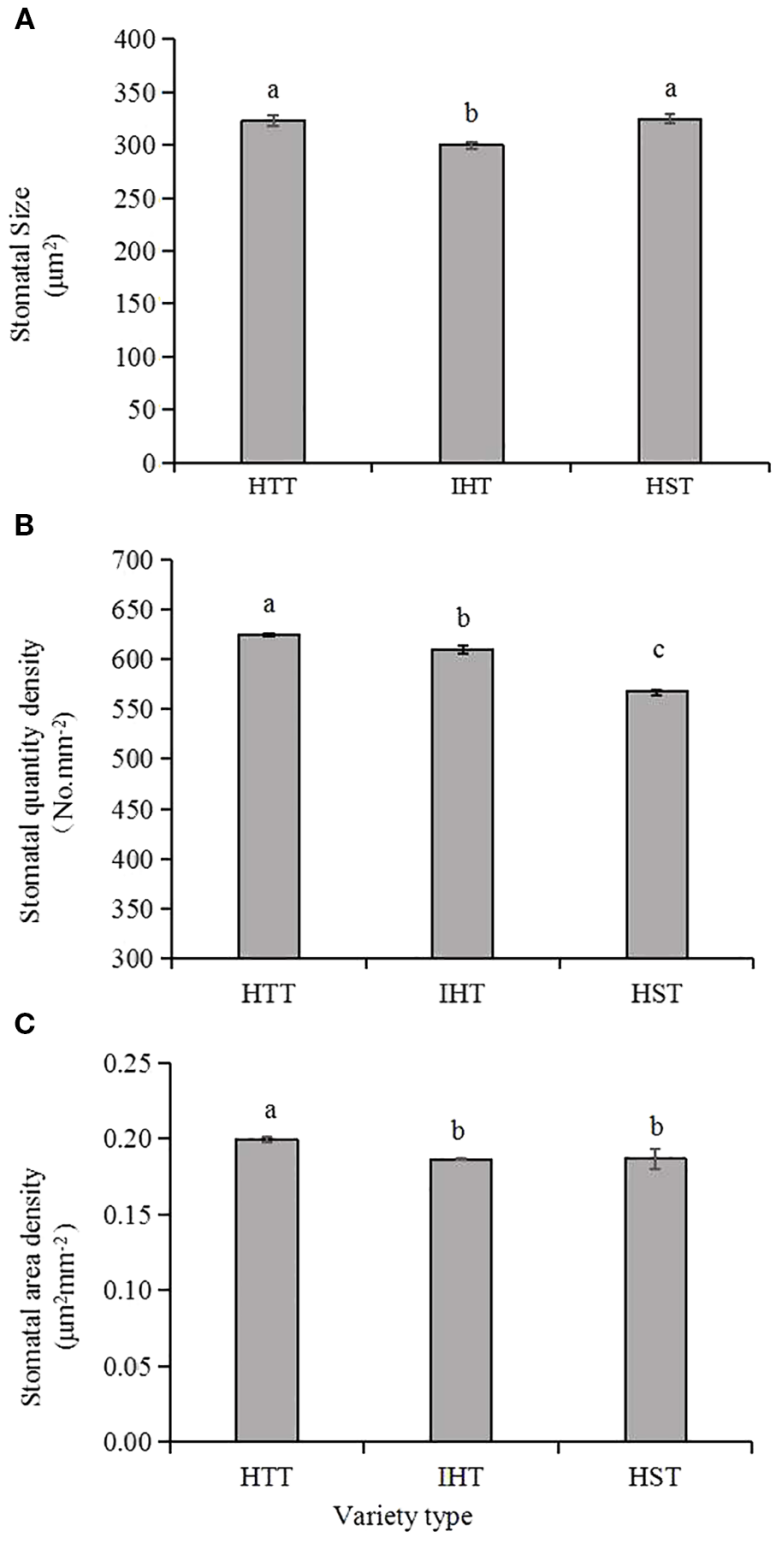
Comparison of stomatal characteristics in the three types of rice varieties responding to heat. HTT, heat-tolerant type; IHT, intermediate heat-tolerant type; HST, heat-susceptible type. Different lowercase letters above each bar represent statistically significant differences (P<0.05) between varietal types. **(A–C)** show the stomatal size, stomatal quantity densities and the stomatal area densities of the three types of the 28 varieties, respectively.

**Figure 5:**
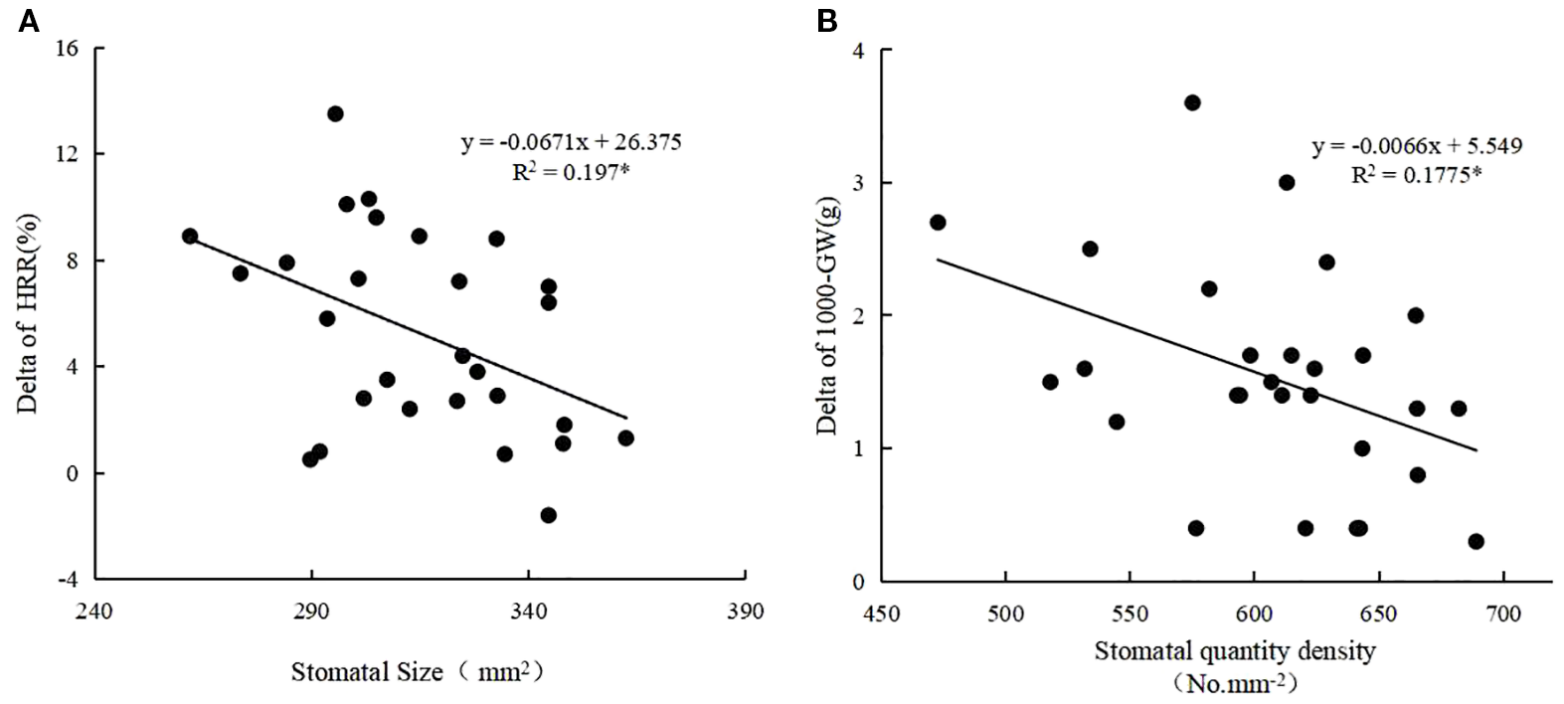
Correlation analysis of stomatal density and size with rice delta-value (Crop2-Crop1) of HRR (head rice rate) **(A)**, GW(1,000-grain weight) **(B)**, respectively. The R value with * means the correlation between the two factors is significant at P<0.05. n = 28.

Generally, stomatal area density ranged from 0.15 μm^2^ mm^−2^ to 0.23 μm^2^ mm^−2^ (average 0.19 μm^2^ mm^−2^), stomatal quantity density ranged from 472.7 mm^2^ to 689.1 mm^2^ (average 610.7 mm^2^) and stomatal size ranged from 273.6 μm^2^ to 364.9 μm^2^ (average 314.8 μm^2^) (**Table 4**). Heat-tolerant varieties had the highest stomatal quantity densities, followed by intermediate varieties, while susceptible varieties had the lowest stomatal area density. Similarly, heat-tolerant varieties had the highest stomatal area densities, while heat-intermediate and heat-susceptible varieties had similar but lower values (**Figure 4**). Correlation analyses revealed significant negative correlations between stomatal size and heat sensitiveness of all varieties (expressed as the delta of head rice rate), as well as between stomatal quantity density and heat sensitivity of all varieties (expressed as the delta of 1,000-grain weight) as well (**Figure 5**).

**Table 4:** Some stomatal parameters of rice varieties.

Varieties	Quantity density (No. mm^−2^)	Area density (μm^2^ mm^−2^)	Stomatal size (µm^2^)
Qunyou 1256	629.1 ± 26.8	0.19 ± 0.01	295.5 ± 25.0
Yexiangyousimiao	581.8 ± 6.2	0.19 ± 0.01	303.2 ± 16.4
Shuijing 3	472.7 ± 25.1	0.16 ± 0.01	332.6 ± 13.3
Qunyou 2	533.9 ± 26.8	0.17 ± 0.02	324.0 ± 26.6
Yuzhenxiang	613.0 ± 26.4	0.21 ± 0.03	344.6 ± 12.5
Huruan 1212	575.1 ± 25.1	0.21 ± 0.02	348.2 ± 6.7
Chuanyou6203	642.3 ± 3.6	0.19 ± 0.01	298.1 ± 9.0
Yexiangyouxinhuazhan	620.5 ± 27.2	0.19 ± 0.01	293.6 ± 8.1
Xiligongmi	665.6 ± 28.4	0.22 ± 0.01	324.8 ± 21.5
Changtianyou 9	517.9 ± 15.5	0.18 ± 0.02	304.9 ± 4.6
Baixiangyou 9978	598.3 ± 27.0	0.18 ± 0.02	314.8 ± 6.6
Baixiangyou 125	665.3 ± 25.1	0.18 ± 0.00	262 ± 8.5.0
Shennongyou 228	593.0 ± 24.4	0.17 ± 0.02	284.3 ± 16.1
Guiyu 8	624.1 ± 20.3	0.17 ± 0.03	273.6 ± 31.7
Yexiangyoulisi	544.6 ± 21.2	0.16 ± 0.01	300.8 ± 11.1
Yujing 91	622.6 ± 14.7	0.22 ± 0.01	344.6 ± 21.1
Taixiangyouyuesimiao	682.1 ± 11.5	0.22 ± 0.02	328.2 ± 25.9
Yuxiang 203	594.1 ± 30.8	0.18 ± 0.02	307.4 ± 27.9
Taiyou 398	606.8 ± 12.1	0.2. ± 0.02	332.8 ± 23.6
Guiyu 9	643.6 ± 11.0	0.19 ± 0.01	302.0 ± 13.0
Nongxiang 32	664.8 ± 13.5	0.22 ± 0.02	323.5 ± 31.6
Exiang 2	531.7 ± 10.1	0.18 ± 0.01	347.9 ± 20.6
Wanxiangyou 982	614.8 ± 12.0	0.18 ± 0.01	291.9 ± 18.3
Taoyouxiangzhan	611.0 ± 11.4	0.19 ± 0.02	334.5 ± 17.2
Taiyou 871	641.1 ± 15.9	0.19 ± 0.03	312.6 ± 18.9
Yixiangyou 2115	689.1 ± 19.1	0.24 ± 0.02	362.4 ± 16.7
Taiyou 98	576.5 ± 21.7	0.17 ± 0.01	289.7 ± 6.6
Jiafengyou 2	643.3 ± 11.1	0.22 ± 0.00	344.6 ± 10.4

## 4 Discussion

In recent years, the impact of climate change on agriculture has attracted growing attention (He et al., 2022; Wang et al., 2025; Liu et al., 2023; Liu et al., 2020a; Muleke et al., 2023). Also, heat at grain filling and its importance in rice have also drawn unprecedented attention. Gong noted that the grain filling stage was the most critical period during which temperature affected rice quality (Gong et al., 2013). Yamakawa and Hakata reported that high temperatures shortened grain filling, hindered the accumulation and transport of photosynthates, and reduced the activity of sucrose–starch metabolic enzymes in grains (Yamakawa and Hakata, 2010). Heat stress have been reported to decrease rice yield and reduce rice quality (Krishnan et al., 2011). Okpala et al. found that the grain quality of rice grown under high temperature stress deteriorated faster during storage compared to rice grown under low temperature conditions (Okpala et al., 2020). According to China’s latest standards for high-quality rice (NBQTC, 2017), such varieties have low chalkiness, high head rice rate, limited amylase content (14%–24%), and high sensory evaluation value. Except for amylose content, the other three indicators may deteriorated under heat-forced ripening.

Previous studies have revealed several mechanisms of varietal tolerant to heat during flowering, including lower canopy temperatures in tolerant varieties compared to heat-susceptible ones (Jagadish et al., 2010; Julia and Dingkuhn, 2013; Matsui et al., 2005, 2007; Ishimaru et al., 2009). It has been shown that transpiration can lead to evaporative cooling in rice, which might contribute to varietal tolerance to heat at flowering (Yoshimoto et al., 2022; Jones, 1999; Tian et al., 2024). In this study, through systematic measurements, we showed that rice evaporative cooling derived from transpiration effect played a pivotal role in controlling canopy temperature during grain filling, similar to that observed at flowering. Further, we found that heat-tolerant varieties had the highest stomatal conductance, followed by heat-intermediate varieties, while heat-susceptible varieties had the lowest stomatal conductance.

It appears that at high temperature, stomata can control transpiration rates through their density distribution and overall stomatal conductance, there by regulating panicle and floret temperatures during grain filling (Bertolino et al., 2019; Franks et al., 2009). In this experiment, when measuring stomatal-related crop traits under field conditions, we not only monitored real-time stomatal conductance and leaf or panicle temperatures in different varieties but also measured stomatal morphological characteristics. We found that varieties with higher stomatal quantity density and greater stomatal size tended to be more tolerant to heat. Meanwhile, we noticed that although some stomatal morphological characteristics had significant correlation with varietal tolerance at grain filling, the determination coefficient (r2) was quite small, suggesting that other factors might influence the relationship, such as heat shock protein expression, accumulation of reactive oxygen species (ROS) (Li et al., 2024), and transmission of calcium ion signals (Qiao et al., 2015; Gao et al., 2022) Moreover, the reaction of stomatal structure to temperature appears to be somewhat complicated among the varieties. Thus, further studies focusing on this area are necessary.

In breeding research, stomatal conductance, panicle temperature, and canopy temperature are all considered ‘dynamic indicators’ in the field, where the measurements are subject to time and weather restrictions, as well as inevitable fluctuations caused by environmental conditions. Conversely, stomatal morphological characteristics are ‘static indicators,’ which are less affected by environmental conditions and can be measured more quickly and easily. Therefore, rice breeders may use such attributes instead of panicle and leaf temperature to screen for genotypes with enhanced capacity for heat avoidance. Our results suggest that stomatal quantity density and stomatal size may be useful attributes for genetic screening.

## 5 Conclusions

In the present study, we showed that high-quality rice varieties grown under heat stress were widely affected by heat-forced ripening. Under heat stress, compared with heat-susceptible varieties, the leaf and panicle temperatures of heat-tolerant varieties decreased significantly, and they maintained higher stomatal conductance during grain filling, which was characterized by higher stomatal quantity and area densities. The results presented in this study provide useful insights into the traits to be selected by rice breeders working to improve rice grain quality and yield under heat stress.

## Data Availability

The raw data supporting the conclusions of this article will be made available by the authors, without undue reservation.
